# Genetic control and prospects of predictive breeding for European winter wheat’s Zeleny sedimentation values and Hagberg-Perten falling number

**DOI:** 10.1007/s00122-023-04450-7

**Published:** 2023-10-24

**Authors:** Quddoos H. Muqaddasi, Roop Kamal Muqaddasi, Erhard Ebmeyer, Viktor Korzun, Odile Argillier, Vilson Mirdita, Jochen C. Reif, Martin W. Ganal, Marion S. Röder

**Affiliations:** 1 European Wheat Breeding Center, BASF Agricultural Solutions GmbH, Am Schwabeplan 8, 06466 Stadt Seeland OT Gatersleben, Germany; 2 Leibniz Institute of Plant Genetics and Crop Plant Research (IPK), Corrensstraße 3, 06466 Stadt Seeland OT Gatersleben, Germany; 3 KWS Lochow GmbH, 29303 Bergen, Germany; 4 KWS SAAT SE & Co. KGaA, 37574 Einbeck, Germany; 5 Syngenta, 28008 Chartres Cedex, France; 6 TraitGenetics GmbH, Am Schwabeplan 1B, 06466 Stadt Seeland OT Gatersleben, Germany; 7 Present Address: KWS SAAT SE & Co. KGaA, Einbeck, 37574 Germany

## Abstract

**Key message:**

Sedimentation values and falling number in the last decades have helped maintain high baking quality despite rigorous selection for grain yield in wheat. Allelic combinations of major loci sustained the bread-making quality while improving grain yield. *Glu-D1*, *Pinb-D1*, and non-gluten proteins are associated with sedimentation values and falling number in European wheat.

**Abstract:**

Zeleny sedimentation values (ZSV) and Hagberg-Perten falling number (HFN) are among the most important parameters that help determine the baking quality classes of wheat and, thus, influence the monetary benefits for growers. We used a published data set of 372 European wheat varieties evaluated in replicated field trials in multiple environments. ZSV and HFN traits hold a wide and significant genotypic variation and high broad-sense heritability. The genetic correlations revealed positive and significant associations of ZSV and HFN with each other, grain protein content (GPC) and grain hardness; however, they were all significantly negatively correlated with grain yield. Besides, GPC appeared to be the major predictor for ZSV and HFN. Our genome-wide association analyses based on high-quality SSR, SNP, and candidate gene markers revealed a strong quantitative genetic nature of ZSV and HFN by explaining their total genotypic variance as 41.49% and 38.06%, respectively. The association of known *Glutenin* (*Glu-1*) and *Puroindoline (Pin-1)* with ZSV provided positive analytic proof of our studies. We report novel candidate loci associated with globulins and albumins—the non-gluten monomeric proteins in wheat. In addition, predictive breeding analyses for ZSV and HFN suggest using genomic selection in the early stages of breeding programs with an average prediction accuracy of 81 and 59%, respectively.

**Supplementary Information:**

The online version contains supplementary material available at 10.1007/s00122-023-04450-7.

## Introduction

Wheat producers and downstream value chains place a high premium on grain’s baking quality because it affects the end-user value and yields high monetary profits. Baking quality, therefore, becomes an important selection criterion in wheat breeding programs across the globe. In Germany, wheat varieties are released according to different quality classes, and several traits form the basis for determining a given class (Sortenliste 2021). Zeleny sedimentation values (ZSV) and Hagberg-Perten falling number (HFN) are among the most important parameters that help determine the quality class of a given wheat line. During registration trials, the lines are evaluated at two management intensity levels, i.e., without (level-1) and with (level-2) the application of plant growth regulators and fungicides. At level-1, measurements are conducted mainly for the agronomic and disease traits. On the other hand, data gathered at level-2 primarily form the basis for describing quality characteristics. Grain yield (GY) is measured at both intensity levels (Sortenliste 2021). It is known that nitrogen fertilizers strongly impact both grain yield and quality in wheat. However, given the Nitrates Directive (91/676/EEC)—a core legislation to reduce agriculture-based nitrate emissions into water bodies in the European Union—German Fertilizer Ordinance (Düngeverordnung) regulates nitrogen and phosphorous emissions into water bodies (Justiz 2017). Thus, the limited application of organic and inorganic fertilizers to reduce the risks of nutrient emissions warrant the exploitation of genetics and breeding approaches to enhance the genetic gain for grain quality traits for climate-smart agricultural practices. Wheat grain quality is primarily determined by its texture, protein content, and quality. Historically, wheat proteins are divided into four major fractions: glutenin, gliadin, globulins, and albumins (Osborne 1907). Glutenin and gliadin together form “gluten”—the largest natural macropolymer in wheat that affects wheat dough’s viscoelastic properties and the appearance and structure of flour-based products (Lukow et al. 1989; Payne 1987). Globulins and albumins, on the other hand, form the “non-gluten” fraction of wheat proteins, and, although present in minor fraction, their ratio and quality were reported to affect dough quality and flour processing (Gupta et al. 1992; Pence et al. 1954; Zhang et al. 2021).

The Zeleny sedimentation test determines the wheat gluten content and quality whereby the flour is mixed in a lactic acid solution that causes the gluten to expand and sediment. Larger ZSV (slower sedimentation) represents high gluten content, strength, and quality—a means for predicting the bread-making quality of flour (Zeleny 1962). Major high-molecular-weight (HMW) glutenin (*Glu-1*) loci, i.e., *Glu-A1*, *-B1*, and *-D1*, are present on the long arm of group-1 wheat chromosomes (Payne and Lawrence 1983). The low molecular weight (LMW) glutenin loci (*Glu-3*), on the other hand, are *Glu-A3*, *-B3*, and *-D3* that are present on the short arm of group-1 homoeologous chromosomes (Singh and Shepherd 1988). The *Glu-3* loci, consisting of several alleles, were reported to be linked with gliadin (*Gli-1*; *Gli-A1*, *-B1*, and *-D1*) loci (Brown and Flavell 1981). Variations in the haplotypes of gluten loci yield varying gluten content and qualities, consequently determining the overall grain quality. Würschum et al. (2016) recently reported gluten loci’s association with sedimentation values. From the major known loci, the puroindoline (*Pin-1*) genes—present at the *Hardness* locus—which mainly affects the grain texture (Morris 2002), were also associated with ZSV (Kristensen et al. 2018; Mohler et al. 2012; Würschum et al. 2016). In addition to *Glu* and *Pin* loci, peroxidases (PODs)—that are widely distributed in cereals—have also been associated with improved rheological properties of flour doughs, loaf volume, crumb structure, and overall bread making (Geng et al. 2019; van Oort et al. 2000; Zhou et al. 2021).

Hagberg-Perten falling number test indicates starch degrading enzyme α-amylase’s activity in wheat flour (Perten 1964). Higher α-amylase activity quickly breaks down the grain starch particles into glucose and maltose reducing the viscosity of the slurry (mix of wheat flour in distilled water). Consequently, a viscometer (stirrer or plunger) falls rapidly (time measured in seconds) through lesser viscous slurry which results in a lower HFN (shorter time). However, if the starch particles are intact, the viscometer falls slowly through the thick slurry resulting in high HFN. Low HFN (high α-amylase activity) is associated with pre-harvest spouting that imposes a significant negative impact on the grain quality resulting in flour that produces sticky and weaker doughs as well as smaller and deformed bread loaves.

Most grain quality traits harbor quantitative genetic control, i.e., their total genetic variance is controlled by the concerted action of large- as well as small-effect loci. In such a scenario, prediction of the total genetic value based on a large number of marker genotypes conferring both large and small effects on the trait becomes a method-of-choice (Meuwissen et al. 2001). Wheat grain quality traits, e.g., ZSV, HFN, flour water retention, and flour yield, are usually evaluated at the later stages of the breeding programs because, on the one hand, the kernel availability is usually scarce in earlier generations, and, on the other hand, most analyses are usually time- and cost-intensive and, thus, analyzing a large number of candidates becomes a virtual impossibility. Hence, quality data are gathered at the later breeding stages on the lines that show promising agronomic trait values and good disease resistance packages. Genome-wide prediction, by using phenotypic data collected at later stages on a relatively larger set of environments as a “training set,” can help select the promising lines for quality traits in early breeding cycles by predicting their genetic values. In addition, in line and hybrid breeding programs, genome-wide prediction of traits can help design the parental crosses with better genetic gain. Recent studies have shown the promise of genome-wide prediction of quality traits for genomic selection (Kristensen et al. 2018; Liu et al. 2016; Muqaddasi et al. 2020; Sandhu et al. 2021).

Gogna et al (2022) published several agronomic, disease, and quality traits’ phenotypic and genotypic data in the GABI panel of 372 registered European wheat varieties. In this study, we took advantage of the published data on ZSV and HFN plus further traits and analyzed them in more detail. We show the relationships and genetic trends of grain quality traits, especially with grain yield (GY). Moreover, the value of glutenin, puroindoline, and plant height loci is illustrated via constructing haplotypes to breed for sustainable or better GY. Our association analyses revealed the quantitative genetic nature of ZSV and HFN and identified known and novel putative loci annotated as globulins, albumins, and peroxide. These genes affect the quality profiles of wheat grain. We also performed genome-wide predictions for ZSV and HFN to evaluate the prospects of predictive breeding: the results point to a promising use of genomic selection for these traits to increase the genetic gain per unit of time and perhaps cost.

## Material and methods

The phenotypic and genomic datasets used in this study were previously published (Gogna et al. 2022). Therefore, in the following, we have limited ourselves to introducing only the essential information on phenotyping and genotyping.

### Field trials and phenotypic data collection

A European wheat panel (GABI) comprising 372 varieties (358 winter type; 14 spring type) was evaluated for two major grain quality traits viz. Zeleny sedimentation values (ZSV; ml) and Hagberg-Perten falling number (HFN; s). The phenotypic data of these two traits were gathered from eight and four environments, respectively. Each environment was considered as a location-by-year combination. The field trials were conducted using an alpha lattice design with two replications per environment. We did not observe conditions conducive to pre-harvest sprouting (e.g., heterogeneous plots, uneven ripening, and lodging) that can strongly influence HFN. A major reason is that data were gathered by keeping in view the German registration authority’s (Bundessortenamt) practices to obtain quality traits’ data from field trials where standard crop protection and plant growth regulator applications (intensity level-2) are executed: this reduces lodging and consequently impacts quality profiles, including HFN. A further detailed description of field trials, agronomic practices, climatic conditions, and calculation of the adjusted entry means per environment was given previously (Gogna et al. 2022; Zanke et al. 2014). Briefly, the phenotypic measurements for ZSV and HFN were carried out by the collaborating seed companies KWS Lochow GmbH and Syngenta Seeds GmbH by using sample volumes of 400 g grains per harvested field plot based on methods described by the International Association for Cereal Science and Technology (ICC) standard number 116/1 and 107/1, respectively.

### Phenotypic data analyses

The consistency or stability among the environments for both traits was investigated by drawing environment-specific adjusted entry mean values as box-and-whisker plots. To check the general performance of a given trait across environments, we calculated the average correlation by performing Fisher’s $z$ transformation, as described previously (Muqaddasi et al. 2020). Across-environment individual variance components of the genotype, environment, and corresponding residuals were computed based on a restricted maximum likelihood (REML) approach by employing a random model:

1 $$y_{ij} = \mu + g_{i} + e_{j} + \varepsilon_{ij}$$

where $y_{ij}$ is the phenotypic value (adjusted entry mean) of the $i^{th}$ genotype in the $j^{th}$ environment, $\mu$ is the common intercept term, $g_{i}$ is the effect of the $i^{th}$ genotype, $e_{j}$ is the effect of the $j^{th}$ environment, and $\varepsilon_{ij}$ is the corresponding residual term as $\varepsilon \sim N\left( {0,I\sigma_{\varepsilon }^{2} } \right)$ with $I$ and $\sigma_{\varepsilon }^{2}$ being the identity matrix and residual variance, respectively. The broad-sense heritability ($H^{2}$) across environments was calculated as follows:

2 $$H^{2} = \frac{{\sigma_{g}^{2} }}{{\sigma_{g}^{2} + \left( {\frac{{\sigma_{\varepsilon }^{2} }}{nE}} \right)}}$$

where $\sigma_{g}^{2}$ and $\sigma_{\varepsilon }^{2}$ denote the variance components of the genotype and residuals, respectively, and $nE$ represents the number of environments. The best linear unbiased estimations (BLUEs) were computed accordingly by setting the genotype as the fixed effect and all other effects as random in Eq. 1.

### Correlations, genetic trend, and determination of best-fit path analysis model

Since grain protein content (GPC; %), grain hardness (GH; %), and grain yield (GY; dt ha^−1^) are considered during the variety registration trials, we retrieved their phenotypic data (i.e., genotypic values or BLUEs) from a previously conducted study based on multiple environments (Muqaddasi et al. 2020). We calculated the genetic correlations among all five traits based on their across-environments BLUEs, as described previously (Muqaddasi et al. 2020).

To test the genetic trend or progress $(\updelta )$, we performed linear regression based on BLUEs calculated from Eq. 1 and the year-of-registration of only winter wheat varieties (*n* = 325) covering three decades starting from the year 1980 as $\updelta =\mathrm{b}/{y}_{0}$, where $\mathrm{b}$ and ${y}_{0}$ denote the regression slope and the mean of varieties released in 1980, respectively. In addition, we highlighted *Rht-D1* alleles (i.e., tall, and short) in scatterplots to show their distribution across the years of registration.

Path analysis—an extension of multiple regression—allows determining the best-fit hypothesis to explain the relationships between the dependent and independent traits by comparing different models (Streiner 2005). We set GY, ZSV, and HFN as dependent traits and examined two different models as follows:

3 $${\text{Model}}\;{1}:\left\{ {\begin{array}{*{20}l} {GY_{{T_{5} }} \sim GPC_{{T_{1} }} + GH_{{T_{2} }} + ZSV_{{T_{3} }} + HFN_{{T_{4} }} } \hfill \\ {ZSV_{{T_{3} }} \sim GPC_{{T_{1} }} + GH_{{T_{2} }} + HFN_{{T_{4} }} } \hfill \\ {HFN_{{T_{4} }} \sim GPC_{{T_{1} }} + GH_{{T_{2} }} } \hfill \\ \end{array} } \right.$$

4 $${\text{Model}}\;{2}:\left\{ {\begin{array}{*{20}l} {GY_{{T_{5} }} \sim GPC_{{T_{1} }} } \hfill \\ {ZSV_{{T_{3} }} \sim GPC_{{T_{1} }} + GH_{{T_{2} }} } \hfill \\ {HFN_{{T_{4} }} \sim GPC_{{T_{1} }} + GH_{{T_{2} }} } \hfill \\ \end{array} } \right.$$

In model 1 (Eq. 3), all the possible relationships among the investigated traits were exploited. For example, GY was predicted by GPC, GH, ZSV, and HFN; ZSV by GPC, GH, and HFN; and HFN by GPC and GH. The indices, namely χ^2^ test, comparative fit index (CFI), Tucker–Lewis’ index (TLI), and standard root mean square residual (SRMR) were used to analyze the goodness of fit of the model as χ^2^ $\left( P \right) > 0.05$, CFI ≥ 0.90, TLI ≥ 0.95, and SRMR ≤ 0.08 (Suhr 2008). In model 1, some trait relationships were observed to be non-significant which led us to develop model 2 (Eq. 4), in which only significant relationships from model 1 were fitted.

To calculate the effect of the independent traits on the dependent, let $p$ be the path from one trait to another and *T* be the trait number. In the first step of model 2 (Eq. 4), GY was only affected by GPC; therefore, the direct (or total) effect for GY was calculated as $GY_{{T_{5} }} \sim GPC_{{[p_{51} T_{1} ]}}$; whereas, ZSV and HFN were predicted by both GPC and GH. Based on the best-fit indices, model 2 was selected to calculate the effects of each independent trait on the dependent traits (for more details, see the Results section).

### Genotyping and molecular data analyses

The whole wheat panel (*n* = 372) was first genotyped with 732 simple sequence repeat (SSR) markers that resulted in scorable 770 loci and 3176 alleles (Kollers et al. 2013). In addition, two state-of-the-art high-density single nucleotide polymorphic (SNP) marker arrays, namely, 90,000 Illumina iSelect (Wang et al. 2014) and 35,000 Axiom Affymetrix Breeders Array (Allen et al. 2017) were employed. The genetic (cM) for SSRs were retrieved from Sorrells et al. (2011) while the physical positions (bp) for SNP arrays were retrieved from Sun et al. (2020).

Since high-molecular-weight glutenin subunits (HMW-GS; *Glu-1*) and grain hardness puroindoline (*Pin-1*) genes are reported to have a significant impact on wheat end-use quality, we sequenced the whole panel to observe their allelic frequency and impact in European wheat. Each *Glu-1* locus on chromosomes 1AL (*Glu-A1*), 1BL (*Glu-B1*), and 1DL (*Glu-D1*) consists of two tightly linked genes encoding x- and y-type HMW-GS. PCR-based candidate/functional markers were applied to distinguish the wheat varieties for null-, x-, and y-type glutenin subunits. For *Glu-A1*, as described in Liu et al. (2008), the functional marker UMN19 was used to distinguish between Ax-null and Ax2* subunits. For *Glu-B1*, two markers were used: one distinguished between Bx6 and Bx7 or Bx17 subunits (Schwarz et al. 2004), and the second distinguished between Bx7^NE^ (normally expressed) and Bx7^OE^ (overexpressed) subunits (Butow et al. 2003). For *Glu-D1*, UMN25 and UNM26 functional markers helped discriminate Dx2 + Dy12 and Dx5 + Dy10 subunits (Liu et al. 2008).

For *Pin-1* loci, i.e., *Pina-D1* and *Pinb-D1*, pyrosequencing technology was employed to genotype the whole panel for the frequently present alleles in European wheat, viz., *Pina-D1a*, *Pina-D1b*, *Pinb-D1a*, *Pinb-D1b*, *Pinb-D1c*, and *Pinb-D1d*. The details about pyrosequencing were given previously by Huang and Röder (2005). As Morris (2002) described, the presence to both *Pin* wild type alleles, i.e., *Pina-D1a* and *Pinb-D1a*﻿, leads to soft endosperm texture, whereas loss-of-function mutations in any of the two loci lead to wheats with hard endosperm. We divided the whole panel based on soft and hard wheats as boxplots and analyzed the difference between the means of both categories via Welch’s two-sided *t*-test by assuming unequal variances at the confidence interval of 0.95.

Introduction of reduced height (*Rht*) genes resulted in significant improvement in GY in wheat (Hedden 2003) and, given the negative relationship between GY and quality traits, the whole panel was genotyped with *Rht-D1* to observe its allelic effect on GY and grain quality traits (Muqaddasi et al. 2017).

For all candidate markers (i.e., *Glu-D1*, *Rht-D1*, and *Pin-1*), boxplots were drawn for the complete set of varieties (BLUEs) and those harboring reference and variant alleles. We used Welch’s two-sided *t*-test to observe if there existed significant differences between the means of varieties harboring different alleles.

### Genome-wide association studies and candidate gene identification

We performed genome-wide association studies (GWAS) based on the BLUEs calculated across environments and molecular markers that passed the quality criteria of < 5% missing values and > 5% minor allele frequency. We combined the SSRs, SNP arrays, and functional-gene markers for the molecular markers set. Following Yu et al. (2006), a standard linear mixed-effect model was used to perform GWAS as:

5 $$y = 1\mu + X\beta + Pv + Zu + \varepsilon$$

where *y* is the column vector of BLUEs of each genotype calculated across environments, $\mu$ is the common intercept, $\beta ,{ }v,{ }u$ and $\varepsilon$ are the vectors of marker, population structure (principal components), polygenic background, and residual error effects, respectively; *X*, *P* and *Z* are the corresponding design matrices. $\mu ,\tau ,{ }\beta ,$ and *v* were assumed to be fixed while *u* and $\varepsilon$ as random with $u\sim N\left( {0,G\sigma_{u}^{2} } \right)$ and $\varepsilon \sim N\left( {0,I\sigma_{\varepsilon }^{2} } \right)$. The variance-covariance genomic relationship matrix (*G*) was calculated based on the second solution, as described by VanRaden (2008).

To declare the marker-trait associations (MTA), we employed the Bonferroni correction $\left( {\upalpha } \right)$ criterion to account for multiple testing as ${\upalpha } = - \log_{10} \left( {\frac{0.1}{p}} \right)$ where *p* is the number of quality markers used in the GWAS. Since Bonferroni’s correction amounted to $- \log_{10} \left( P \right) = 5.34$, we used an exploratory threshold of $- \log_{10} \left( P \right) = 3.5$ to identify the MTA. As described by Utz et al. (2000), the genotypic variance ($p_{G}$) explained by all the QTL was determined as $p_{G} = \left( {\frac{{R_{{{\text{adj}}}}^{2} }}{{H^{2} }}} \right) \times 100$ where $R_{adj}^{2}$ was calculated as $R_{{{\text{adj}}}}^{2} = R^{2} - \left( {\frac{{z^{\prime}}}{{N - z^{\prime} - 1}}} \right)\left( {1 - R^{2} } \right)$ by fitting the MTA $\left( {z^{\prime}} \right)$ in the order of their descending *P* values in a multiple linear regression model; *R*^2^, *N* and *H*^2^ denote the regression coefficient, number of observations, and the broad-sense heritability calculated in Eq. 2. The $p_{G}$ explained by the individual MTA was accordingly calculated from their sum of squares.

The genetic gain per unit time and cost become co-extensive with the genotypic variance explained by the trait-associated markers. For example, if the quantitative trait loci (QTL) underlying a given trait explain a small proportion of genotypic variance, marker-assisted selection for these QTL becomes cost and time intensive. Hence, we selected the markers that explained ${p}_{G}$ of  ≥  ~ 10% and designated as the QTL for the respective trait. We considered the markers with the highest $-{\mathrm{log}}_{10}\left(P\right)$ value and ${p}_{G}$ from each QTL as “representative markers” for that QTL. The QTL were named based on accepted QTL naming conventions in wheat literature (Boden et al. 2023). The sequences harboring each SNP (MTA) were taken from the genotyping arrays and BLASTed onto the wheat’s reference sequence to determine their corresponding gene identifiers and human-readable descriptions. Also, the gene-start, -stop, and the SNP position (bp) were retrieved from Sun et al. (2020).

### Genome-wide predictions

We evaluated the genome-wide prediction (GP) accuracies of ZSV and HFN by using five genomic selection (GS) models with different marker variance assumptions. The GS models were genomic best linear unbiased prediction (GBLUP), BayesA, BayesB, BayesC, and reproducing kernel Hilbert space regression (RKHSR) (Gianola and Van Kaam 2008; Habier et al. 2007; Meuwissen et al. 2001; Pérez and Los Campos 2014; VanRaden 2008). The implementation of GBLUP and RKHSR on the same panel has been reported previously (Muqaddasi et al. 2019). For Bayesian implementation (BayesA–C), we followed Pérez and Los Campos 2014 and implemented the same prior densities and default hyperparameters mentioned in the BGLR package.

We evaluated the accuracy $\left({r}_{GP}\right)$ of all prediction models by using a fivefold cross-validation scenario. The varieties were randomly divided into five subsets: four of them were used as the training set to estimate the genetic values of the remaining test set. The accuracy of prediction was defined as Pearson’s product-moment correlation between the observed $\left(y\right)$ and predicted $\left(\widehat{y}\right)$ genetic values standardized by the square root of the broad-sense heritability, as ${r}_{GP}=\frac{cor\left(y, \widehat{y}\right)}{H}$. Since the cross-validation runs were repeated for 50 cycles, the mean and standard deviation values were calculated to show the performance of the individual GS model. Unless stated otherwise, all calculations were performed in software R (Team 2013) mainly by using packages lme4 (Bates et al. 2015), lavaan (Rosseel 2012), and rrBLUP (Endelman 2011).

## Results

### Phenotypic data analyses reveal consistent performance, significant genotypic variation, and stable genetic trend for sedimentation values and falling number

Grain quality traits, viz. Zeleny sedimentation values (ZSV) and Hagberg-Perten falling number (HFN) were assessed on a panel of 372 wheat varieties registered in European markets (Table S1). The field trials to collect data were performed in replicated trials in eight and four environments, respectively. Boxplots based on adjusted entry means drawn for individual environments showed consistent performance of both traits yielding positive average Pearson’s product-moment correlation $({\overline{r} }_{\left(\mathrm{ZSV}\right)}=0.78;{\overline{r} }_{\left(\mathrm{HFN}\right)}=0.44)$ across all the environments (Fig. S1a,b). Grain protein content (GPC) and grain hardness (GH) are high-throughput traits that can be relatively easily scored non-destructively (e.g., via near-infrared reflectance; NIR) especially in an early generation where the kernel availability is usually scarce. We retrieved the genotypic values (BLUEs) for all 372 varieties from a previous study to evaluate if there existed any genetic correlation and trend. Grain yield (GY) is the principal parameter to select in breeding programs. Like GPC and GH, we retrieved the GY genotypic values calculated based on eight environments from a previous study to find its association with quality traits (Muqaddasi et al. 2020).

**Fig. 1:**
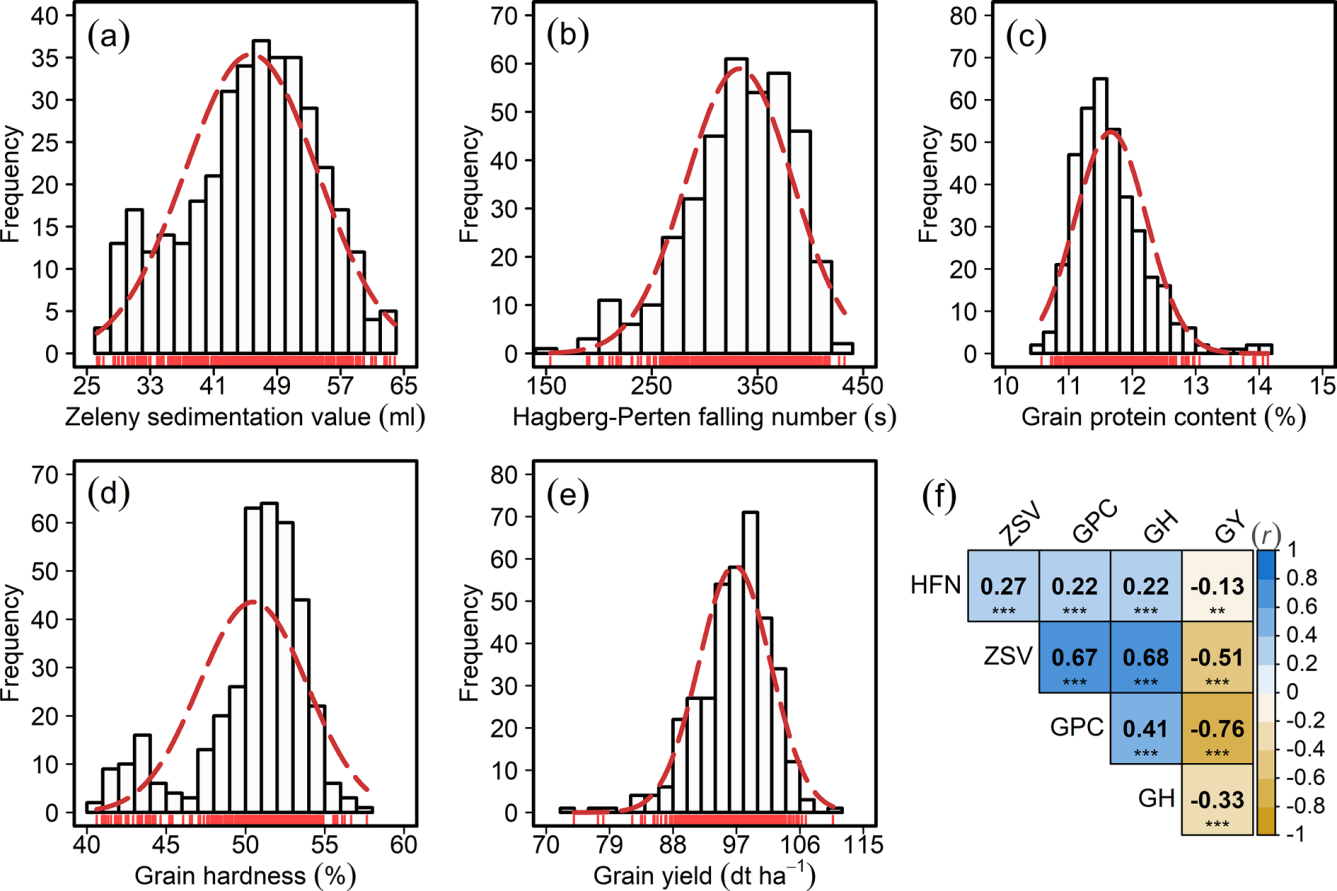
Phenotypic distribution and correlation of the investigated traits in a panel of 372 European wheat varieties. Distribution of (**a**) Zeleny sedimentation value (ZSV), (**b**) Hagberg-Perten falling number (HFN), (**c**) grain protein content (GPC), (**d**) grain hardness (GH), and (**e**) grain yield (GY); (**f**) Pearson’s product-moment correlation $\left(r\right)$ among the investigated traits where *** and ** denote the significance of respective correlation at probability (*P*) of < 0.001 and < 0.01, respectively

Our across years individual variance component analyses revealed wide genotypic variation that was significantly (*P* < 0.001) larger than zero for both ZSV and HFN, with their BLUEs approximating normal distribution (Table 1; Fig. 1a–e). Despite a significant environmental variance, the large genotypic variance translated into high broad-sense heritability estimates of 0.96 and 0.74 for ZSV and HFN, respectively (Table 1). Large genotypic variance coupled with high broad-sense heritability estimates point to, on one hand, a high selection advantage; and, on the other hand, a reasonable underlying allelic diversity that should favor reliable downstream analyses to evaluate promises of genomic approaches.

**Table 1:** Summary statistics of the Zeleny sedimentation values (ZSV) and Hagberg-Perten falling number (HFN) in European wheat varieties

Trait	Minimum	Mean	Maximum	Env	${\sigma }_{G}^{2}$	${\sigma }_{E}^{2}$	${\sigma }_{\varepsilon }^{2}$	${H}^{2}$
ZSV	26.27	45.73	63.83	8	67.68 ^α^	58.19 ^α^	21.01	0.96
HFN	153.93	332.82	432.13	4	1884 ^α^	2267 ^α^	2601	0.74

Env. = number of environments in which the corresponding trait was investigated, ${\sigma }_{G}^{2}$ = genotypic variance, ${\sigma }_{E}^{2}$ = environmental variance, ${\sigma }_{\varepsilon }^{2}$ = residual variance, α = significant at the probability $\left(P\right)$ level of 0.001, ${H}^{2}$ = broad-sense heritability

**Fig. 2:**
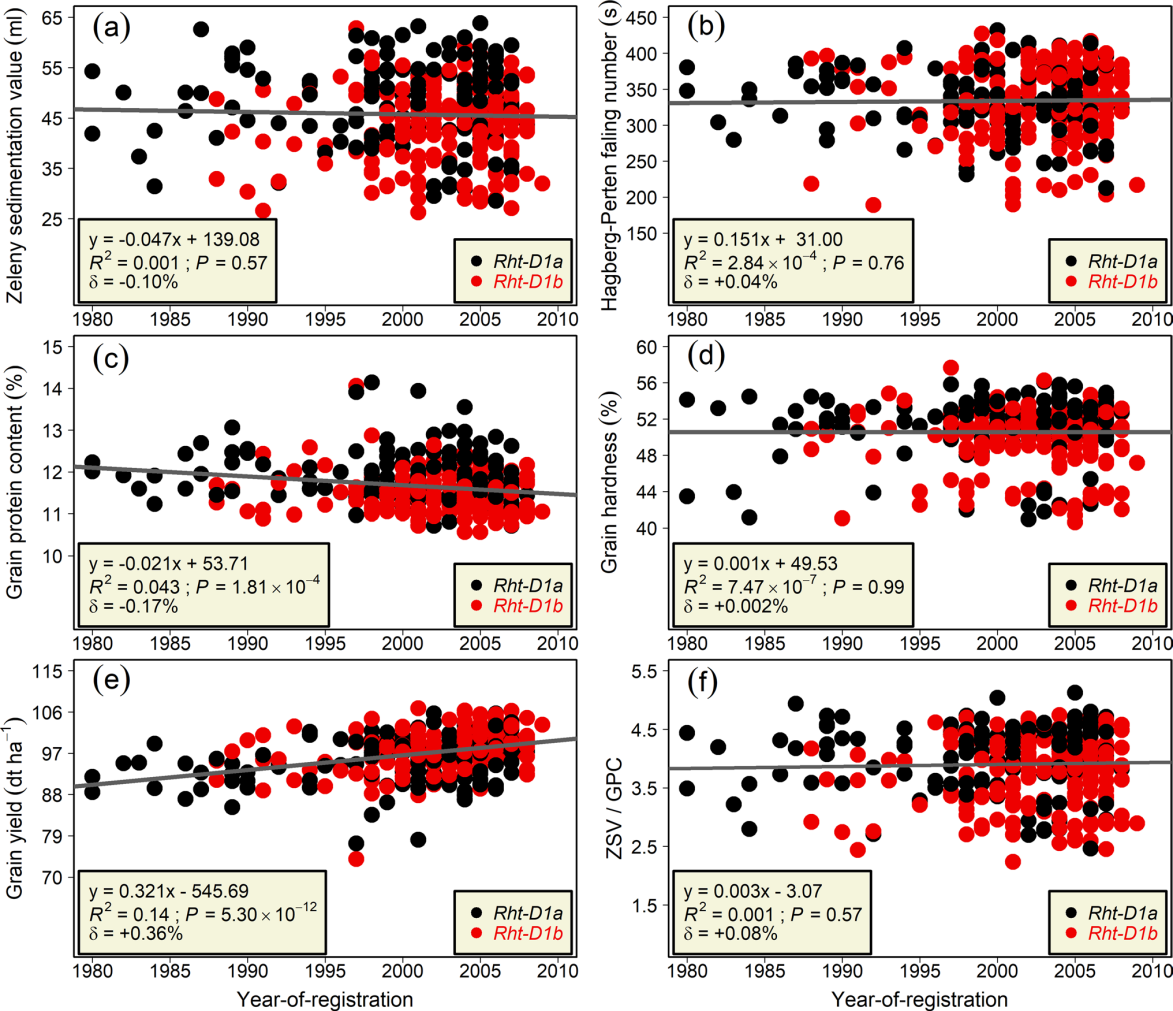
Scatterplot of (**a**) Zeleny sedimentation value (ZSV), (**b**) Hagberg-Perten falling number, (**c**) grain protein content (GPC), (**d**) grain hardness, (**e**) grain yield, and (**f**) ratio of ZSV/GPC as a function of year-of-registration of 325 European winter wheat varieties covering three decades (1980–2009). Regression estimations, coefficient of determination (*R*^2^), significance (*P*) values, and increase per annum (δ) for traits are given in each sub-figure. Black and red dots represent the *Rht-D1a* and *-D1b* alleles, respectively, while the gray solid line represents the linear regression line

Our regression analyses to evaluate the effect of year-of-registration on varieties revealed noteworthy results (Fig. 2a–e). For example, the genetic trend for GPC significantly (*P* < 0.001) decreased by 0.17% per annum (Fig. 2c) whereas GY significantly (*P* < 0.001) increased (0.36%; 0.321 dt ha^−1^y^−1^; Fig. 2e). Statistically non-significant (*P* > 0.05) genetic trends were observed for ZSV, HFN, and GH (Fig. 2a,b,d). In addition to the regression on the absolute trait-BLUEs, we performed the regression on the ratio calculated from ZSV and GPC: these values are indicative of the protein (gluten) quality and content simultaneously. For ZSV/GPC ratio—in contrast to GPC where a negative selection trend was observed—we observed a positive although non-significant genetic trend (Fig. 2e). This suggests that albeit the protein content has decreased, the breeders were able to develop varieties with good protein quality that consistently met baking requirements during registration trials.

### Correlation and path analysis uncover genetic relationships among quality traits and grain yield

The genetic correlation among all five investigated traits based on their BLUEs revealed that all quality traits, viz. ZSV, HFN, GPC, and GH showed a negative correlation with GY. The most pronounced significant negative (− 0.76, *P* < 0.001) correlation was observed between GPC and GY (Fig. 1f). Among the grain quality traits, ZSV showed a higher negative and significant correlation with GPC and GH in comparison with HFN (Fig. 1f). HFN showed significant but lower correlations with all quality as well as GY—this points to a possible different genetic underpinning of ZSV and HFN. Besides, we observed genetic associations among the traits by regressing the quality trait BLUEs on GY: this also showed that (1) the most variance in GY is explained by GPC (*R*^2^ = 0.57) followed by ZSV (*R*^2^ = 0.26), and (2) there, nevertheless, exist varieties with above average values for each combination (Fig. S2a–d).

**Fig. 3:**
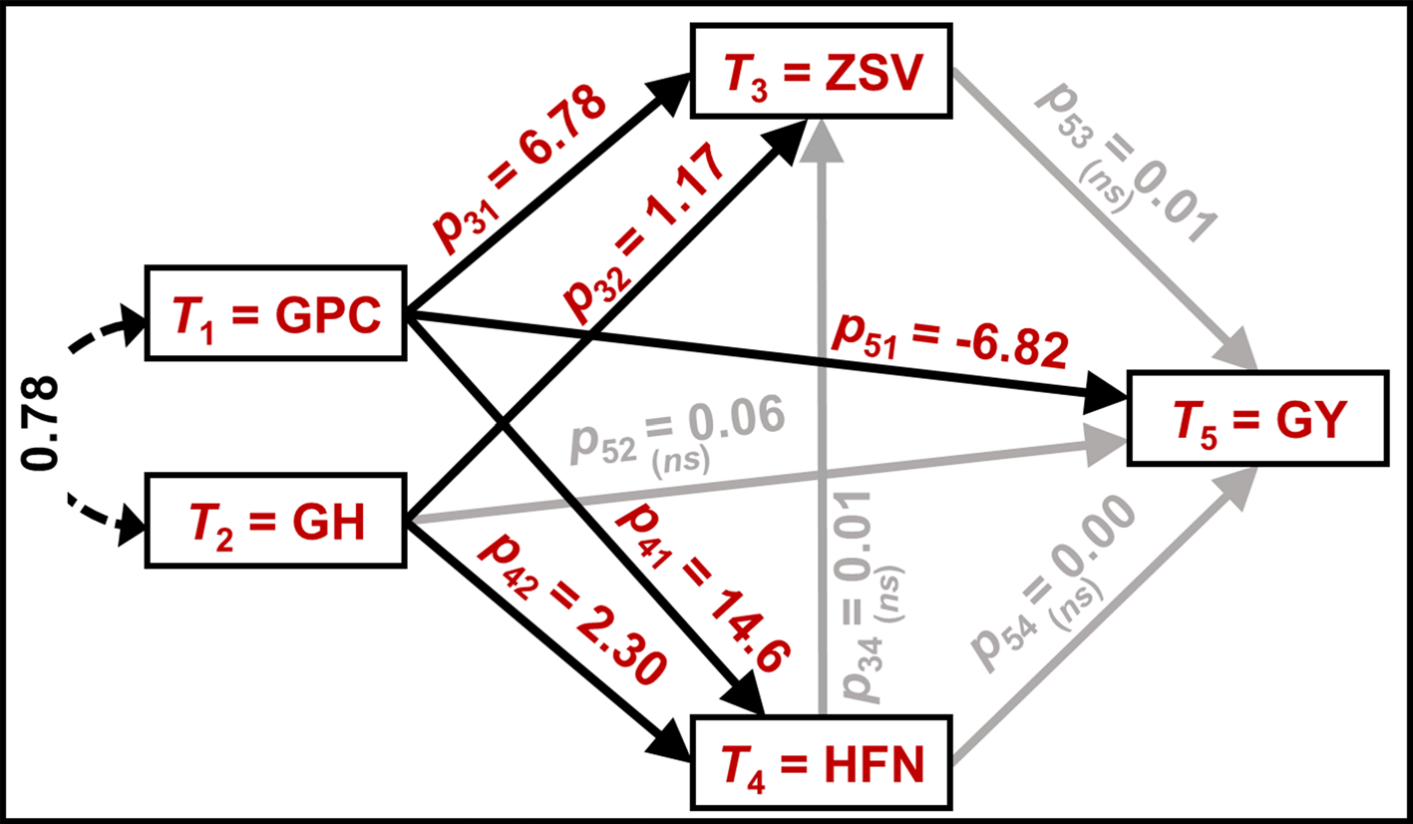
Path diagram elucidating all possible relationships among the investigated traits, viz. Zeleny sedimentation value (ZSV), Hagberg-Perten falling number (HFN), grain protein content (GPC), grain hardness (GH), and grain yield (GY). Single-headed and dashed double-headed curved arrows indicate a trait’s direct effect on another and covariance between the GPC and GH, respectively. The numbers on the single-headed arrows denote the path-coefficients as positive and negative effect of one trait on another. T_1_–T_5_ and *ns* denote the trait numbers and non-significant relationships, respectively

Our path analysis revealed underlying genetic relationships among the grain quality parameters and grain yield (Table S2, Fig. 3) with the most pronounced negative effect of GPC on GY, i.e., every unit increase in GPC resulted in 6.82 units decrease in GY (Fig. 3). In contrast to GY, GPC showed a direct positive effect on ZSV and HFN, i.e., every unit increase in the GPC increased the ZSV and HFN by 6.78 and 14.6 units, respectively. GH displayed positive effects on ZSV and HFN but with overall lower values than GPC. Interestingly, ZSV, HFN, and GH did not significantly affect GY. Interaction between ZSV and HFN was also non-significant (Table S2). These results further cement (1) the possibility of different genetic architecture and interaction of ZSV and HFN, especially concerning GPC, GH, and GY (Fig. 3), and (2) the interpretation of the above-mentioned genetic trend analyses where although the GY increased, except GPC, none of the other quality traits significantly changed.

### GWAS identifies large-effect loci and putative candidate genes for sedimentation values and falling number

**Fig. 4:**
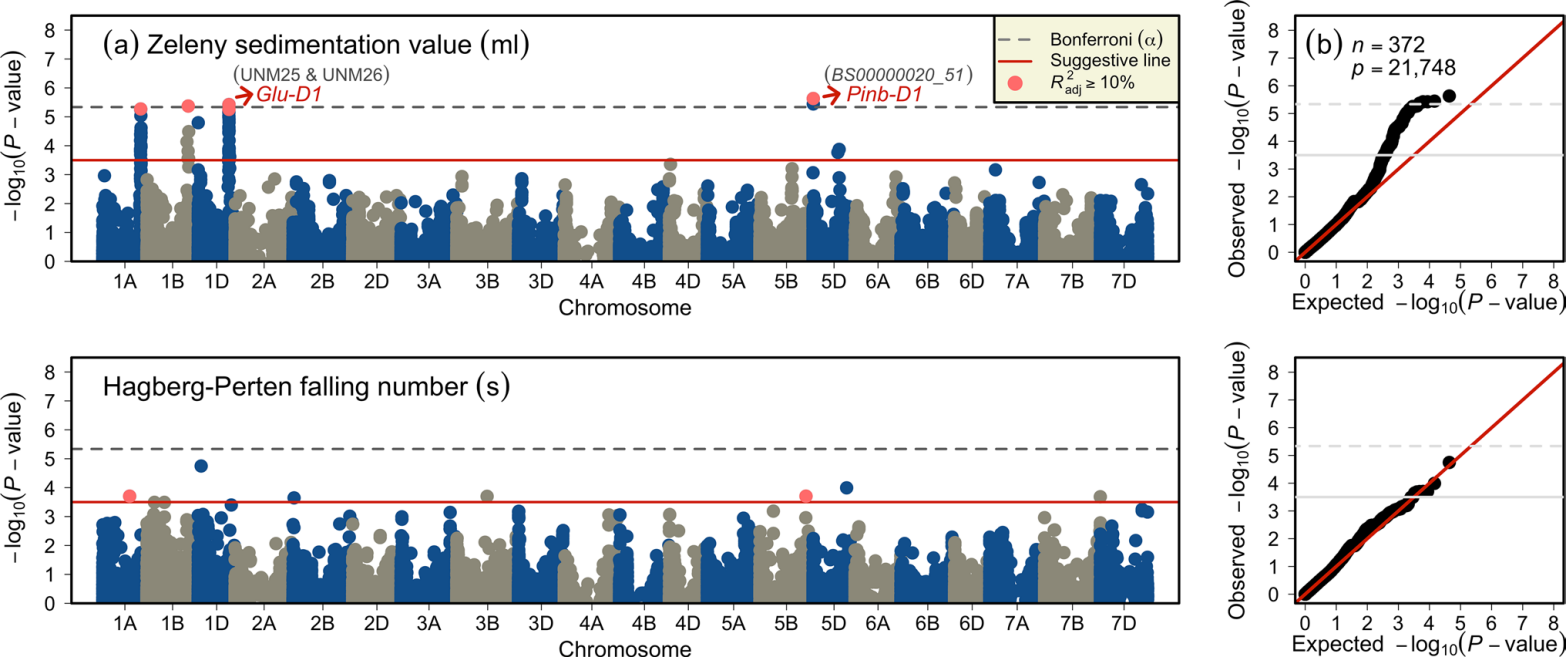
Summary of genome-wide association studies (GWAS) of Zeleny sedimentation values and Hagberg-Perten falling number in a panel of European wheat varieties. (**a**) Manhattan plots show the distribution of marker loci’s significance ${-\mathrm{log}}_{10}(P-\mathrm{value})$ along 21 wheat chromosomes. The dashed gray line indicates the significance threshold based on Bonferroni correction, while red continuous lines represent an exploratory significance threshold. Red dots represent the main-effect QTL’s representative markers for the respective traits. (**b**) Quantile-quantile plots show the distribution of observed *versus* expected (red dashed line) ${-\mathrm{log}}_{10}(P-\mathrm{value})$. *n* and *p* denote the number of varieties and the quality markers used in the GWAS analyses

We elucidated the genetic control of ZSV and HFN based on 372 wheat varieties and genomic markers (Fig. 4). For ZSV, in total, we identified 59 marker-trait associations (MTA) representing four QTL, viz., *QZsv.ipk-1A*, *QZsv.ipk-1B*, *QZsv.ipk-1D*, and *QZsv.ipk-5D* (Tables 2, S3, Figs. 4, S3–S7). However, only seven MTA were identified for HFN, each on separate chromosomes, which signified two QTL with ≥ 10% of genotypic variance as *QHfn.ipk-1A* and *QHfn.ipk-5B* (Tables 2, S3, Figs. 4, S8, S9). Each QTL from ZSV and HFN was represented by the most significant MTA, explaining the largest genotypic variance. The total genotypic variance explained by all the MTA for ZSV and HFN amounted to 41.49% and 38.06%, respectively.

**Table 2:** Quantitative trait loci (QTL) associated with Zeleny sedimentation value (ZSV) and Hagberg-Perten falling number (HFN) in European wheat varieties

QTL	Representative marker	Chr	Pos	$|{\mathrm{log}}_{10}(P)|$	${p}_{G}$
*QZsv.ipk-1A*	*AX-95236907*	1A	509.0	5.27	26.14
*QZsv.ipk-1B*	*AX-95017670*	1B	555.3	5.37	26.35
*QZsv.ipk-1D*	*Glu-D1* (UMN25 & UMN26)	1D	–	5.42	25.56
	*BS00022768_51*	1D	412.2	5.19	26.05
*QZsv.ipk-5D*	*BS00000020_51*	5D	3.6	5.63	11.39
*QHfn.ipk-1A*	*AX-94430348*	1A	360.5	3.70	12.74
*QHfn.ipk-5B*	*wsnp_Ku_rep_c71565_71299640*	5B	618.1	3.70	9.38

Chr. = chromosome, Pos. = Mbp position of the corresponding markers, $|{\mathrm{log}}_{10}(P)|$ = significance value of the corresponding marker, ${p}_{G}$ = percentage of genotypic variance (adjusted *R*^2^)

The MTA BLASTed onto the wheat reference sequence resulted in identifying high-confidence genes. The functional annotations (human-readable descriptions) yielded highly plausible candidate genes. For example, a SNP *AX-95236907* representing the ZSV-QTL *QZsv.ipk-1A* corresponded to the gene *TraesCS1A02G317900* with functional annotation of Peroxidase—an enzyme that is known to influence the wheat quality (Table S3, Fig. S3). For *QZsv.ipk-1D*, the functional marker for the glutenin gene *Glu-D1* (UMN25 and UMN26) that distinguished between Dx5 + DY10 and Dx2 + Dy12 subunits was significantly associated with the ZSV and explained 25.56% of the genotypic variance (Fig. S5). The QTL *QZsv.ipk-1D* was also represented by the marker *BS00022768_51* (gene ID: *TraesCS1D02G317300*) that explained 26.05% of the genotypic variance with functional annotation of globulin—a non-gluten wheat protein that is associated with wheat quality (Table S3, Fig. S6). *QZsv.ipk-5D*, represented by *BS00000020_51* (*TraesCS5D02G004300*), explained 11.39% of the genotypic variance and corresponded to Puroindoline-b (*Pinb-D1*)—a known gene at the *Hardness* locus responsible for controlling wheat grain texture (Morris 2002). For HFN, SNP *AX-94430348* (*TraesCS1A02G200400*) representing the *QHfn.ipk-1A* explained 12.74% of the genotypic variance and corresponded to Albumin-2 protein—another non-gluten protein in wheat’s endosperm (Tables 2, S3, Figs. 4,S8).

### Impact of *Glu-1*, *Rht-D1*, and *Pin-1* loci on wheat quality traits and grain yield

**Fig. 5:**
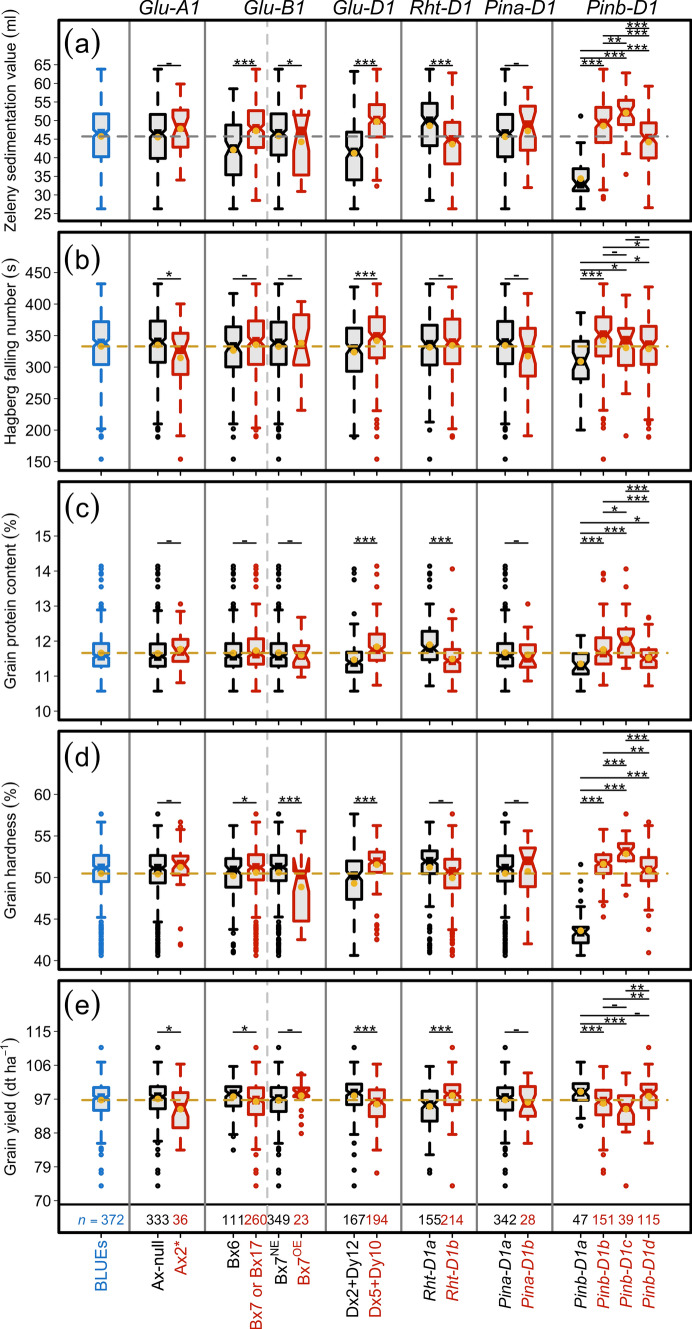
Boxplots showing the allele-wise effect of major loci on (**a**) Zeleny sedimentation value, (**b**) Hagberg-Perten falling number, (**c**) grain protein content, (**d**) grain hardness, and (**e**) grain yield. The header and x-axis represent the gene names and corresponding alleles (subunits), respectively. The first boxplot shows the distribution of best linear unbiased estimations (BLUEs) of each trait. *n* denote the number of varieties in which the corresponding allele was observed. ***, **, *, and – denote the significance values of Welch's two-sided *t*-test at probability (*P*) values of < 0.001, < 0.01, < 0.05, and > 0.05 , respectively

**Fig. 6:**
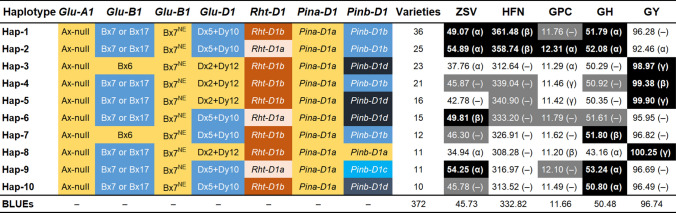
Ten major haplotypes (allelic combinations) based on major loci for glutenins (*Glu*), plant height (*Rht*), and puroindolines (*Pin*), the number of varieties harboring the corresponding haplotype, and trait values based on best linear unbiased estimations (BLUEs) of varieties. The x- and y-type subunits (alleles) are colored for haplotype construction. Welch's two-sided *t-*test was used to observe if there existed significant differences between the means of haplotype-based trait values and total population (*n* = 372). α, β, γ, and – denote the significance value of the *t-*test at the probability (*P*) levels of < 0.001, < 0.01, < 0.05, and > 0.05, respectively. The trait columns (ZSV = Zeleny sedimentation value, HFN = Hagberg-Perten falling number, GPC = grain protein content, GH = grain hardness, and GY = grain yield) are colored as black = values significantly larger than the population mean, gray = values larger but insignificant than the population mean, and white = values lower than the population mean

The entire wheat panel was genotyped for major loci associated with grain quality and yield (*Glu-1*, *Pin-1*, and *Rht-D1*; Table S1, Fig. 5a–e), and several most abundant haplotypes were studied (Fig. 6).

For *Glu-A1*, we used the functional marker UMN19, which separated wheat varieties based on the presence of Ax-null (90.24%) and Ax2* (10.56%) subunits. Welch’s two-sided *t*-test revealed that the presence of Ax-null was associated with significantly (*P* < 0.05) higher values for HFN and GY (Fig. 5b,e); however, for ZSV, GPC, and GH, no significant differences existed (Fig. 5a,c,d).

*Glu-B1*, genotyped with two functional markers that distinguished between (1) Bx6 and Bx7 or Bx17, and (2) Bx7^NE^ and Bx7^OE^ subunits, revealed their frequencies as 29.91, 70.08, 93.82, and 6.18%, respectively. For the first marker, the presence of Bx7 or Bx17 subunit was associated with significantly higher values for ZSV and GH; however, the effect was negative for GY where the varieties harboring Bx7 or Bx17 subunit was associated with decreased (*P* < 0.05) values (Fig. 5e). For HFN and GPC, the difference between the means of varieties harboring these two subunits was non-significant (Fig. 5b,c). The difference between the means of varieties harboring Bx7^NE^ and Bx7^OE^ subunits was not significant for HFN, GPC, and GY (Fig. 5b,c,e); however, the presence of Bx7^OE^ was associated with significantly higher values for ZSV and GH (Fig. 5a,d).

*Glu-D1*—genotyped with the functional markers UMN25 and UMN26—was used to differentiate varieties harboring Dx2 + Dy12 and Dx5 + Dy10 subunits that were present at the frequencies of 46.26 and 53.74%, respectively. *Glu-D1* was observed as one of the most revealing loci: the presence of different subunits showed a highly significant impact on all quality traits as well as GY. For example, the presence of Dx5 + Dy10 subunits, which are known to impact quality traits positively, was associated with significantly (*P* < 0.001) higher values for all quality traits while (Fig. 5a–d). However—as could be deduced from the negative relationship between grain quality and GY—Dx5 + Dy10, in comparison with Dx2 + Dy12, was associated with significantly (*P* < 0.001) lower values for GY (Fig. 5e). The frequencies of these subunits in European varieties, nevertheless, points to the likelihood that breeders generally have been fine-tuning the grain quality traits and GY based on *Glu-D1* in combination with other large-effect genes.

*Rht-D1*—a green revolution gene—is frequently used in European wheat breeding programs to tailor the plant height and improve lodging resistance and GY (Flintham et al. 1997). Thus, from the known negative correlation between GY and grain quality traits, it is expedient to study the effect of tall (*Rht-D1a*) and short (*Rht-D1b*) alleles on quality and GY. The difference between the means of varieties harboring tall (42.01%) and short (57.99%) alleles were non-significant for HFN and GH (Fig. 5b,d) but highly significant (*P* < 0.001) for ZSV, GPC, and GY (Fig. 5a,c,e): *Rht-D1b* presence was associated with reduced values for ZSV and GPC while increased GY.

*Pina-D1*—a gene present at the *Hardness* locus—was represented by wild-type *Pina-D1a* (92.43%) and null *Pina-D1b* (7.57%) alleles in the investigated varieties. However, the allelic influence on varieties was non-significant (Fig. 5). From allelic frequencies, it can also be safely inferred that the breeders in Europe have largely been adjusting the grain texture by exploiting the loci other than *Pina-D1*.

*Pinb-D1* gene was genotyped for four widely present alleles in European varieties, viz., *Pinb-D1a* (13.35%), *-D1b* (42.90%), *-D1c* (11.08%), and *-D1d* (32.67%). Besides *Glu-D1*, *Pinb-D1*’s allelic influence was observed as most pronounced: the use of *Pinb-D1b* and *-D1d* to alter the grain texture in wheat varieties was evident from their frequencies (Fig. 5). The presence of *Pinb-D1a* (wild type) allele was associated with a significant decrease in all investigated quality traits whereas—except for HFN—the higher values were observed for mutant *Pinb-D1c*. As expected, wild-type *Pinb-D1a* and *-D1c* were associated with significantly higher and lower GY values, respectively. Moreover, the *Pinb-D1b*’s presence, compared to *Pinb-D1a*, was associated with significantly higher values for all quality traits while decreasing GY (Fig. 5e): This was less pronounced for *Pinb-D1d* where although the difference between varieties was significant for all quality traits but non-significant for GY (Fig. 6).

**Fig. 7:**
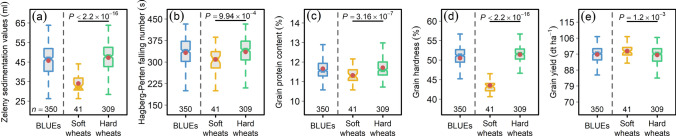
Impact of soft and hard wheats divided according to puroindoline (*Pin*) profiles on (**a**) Zeleny sedimentation value, (**b**) Hagberg-Perten falling number, (**c**) grain protein content, (**d**) grain hardness, and (**e**) grain yield. Soft wheats represent varieties harboring wild-type *Pin* alleles (*Pina-D1a* and *Pinb-D1a*) whereas hard wheats harbor *Pin* allelic variants for one or both *Pina-D1* and *Pinb-D1*. For comparison, the first boxplot within every sub-figure represents the best linear unbiased estimations (BLUEs) across soft and hard wheats. *n* denote the number of varieties present in each category. *P* values denote the significance values of Welch's two-sided *t*-test

Of a total of 372, 350 varieties had complete information for the *Pin-1* loci: 41 harbored soft while 349 hard *Pin* profiles (Table S1). Welch’s two-sided *t*-test revealed that hard wheats harbored significantly (*P* < 0.001) higher values for all the quality traits (Fig. 7a–d) whereas, as expected, we observed a significantly lower values for GY (Fig. 7e).

### Genome-wide prediction accuracy suggests the efficient use of genome-wide selection in wheat breeding programs

**Fig. 8:**
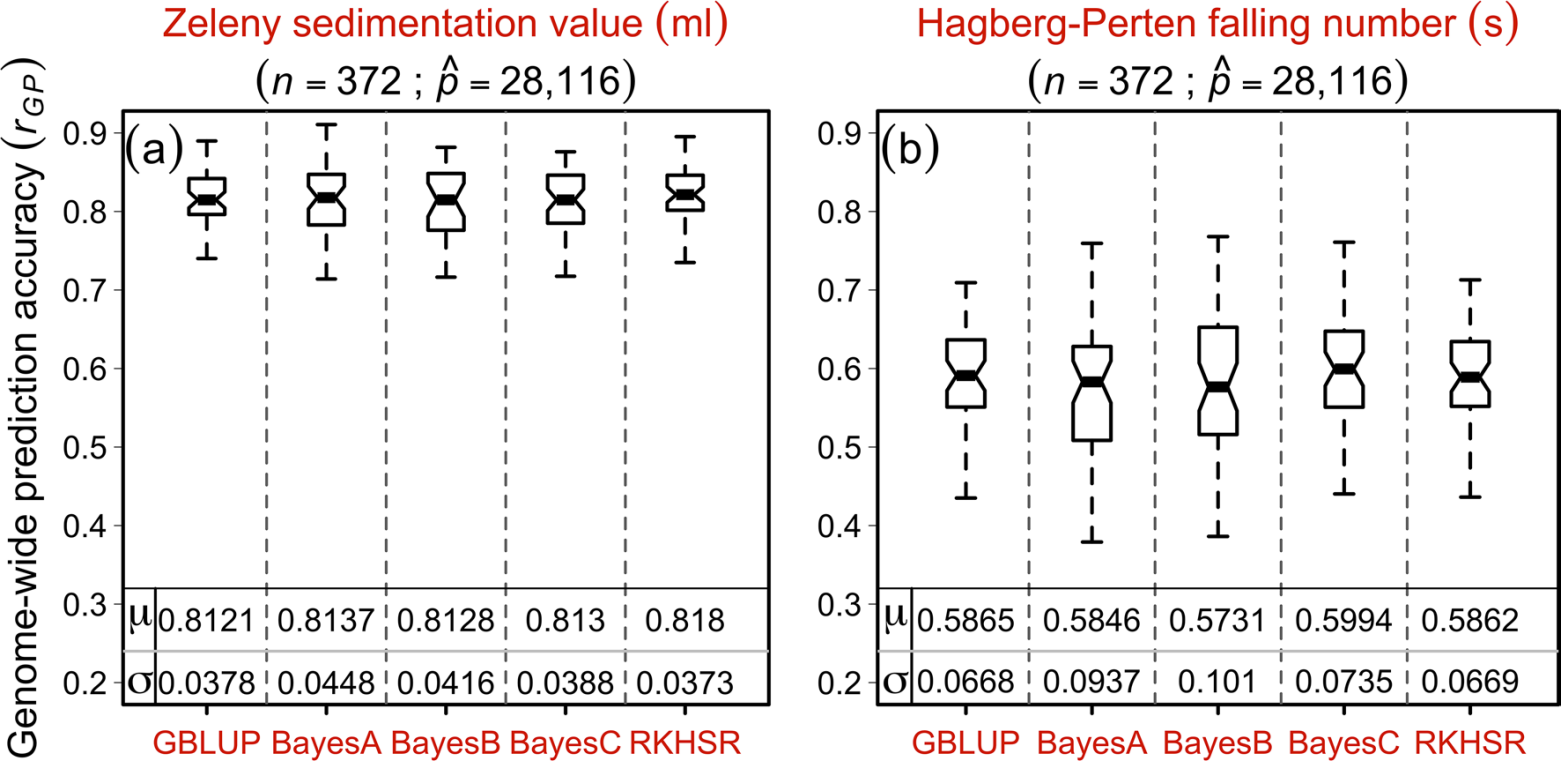
Accuracy of the genome-wide prediction (GP) for (**a**) Zeleny sedimentation values and (**b**) Hagberg-Perten falling number based on five genomic selection models viz. genomic best linear unbiased prediction (GBLUP), BayesA–C, and reproducing kernel Hilbert space regression (RKHSR) evaluated by 50 random runs of fivefold cross-validation cycles. Symbols $\mu$ and $\sigma$ denote the corresponding model's mean prediction accuracy and standard deviation. *n* and $\widehat{p}$ (markers including the unmapped) denote the number of varieties and the quality markers used in the GP analyses

We performed genome-wide predictions to assess the potential of genomics for predictive breeding measured as the correlation between the predicted and observed trait values standardized by the square root of heritability. The mean prediction accuracies resulting from the fivefold cross-validation settings of quality traits produced similar results across all five tested model scenarios, i.e., the GBLUP, Bayes-A, -B, -C, and RKHSR (Fig. 8a,b). It is worth noting that the prediction accuracy for ZSV was ~ 20% (~ 81%) higher than that of HFN (~ 59%): this is consistent with the theory, where prediction accuracies of the traits that are less complex tend to be higher as compared to those with highly complex genetic architecture. RKHSR—mainly used to observe if there exists epistatic interaction among loci—yielded results on par with other models suggesting that epistatic interactions may not be pervasive for investigated traits.

## Discussion

Wheat grain’s baking quality class determines the monetary benefits for growers and, thus, in addition to improved grain yield and disease resistance, fine-tuning and improving the quality trait profiles remains a major target for breeding programs. Most grain quality parameters are influenced by crop management (esp. fertilizer application) practices and the environment. However, there exists variation among wheat varieties that could be ascribed to the underlying genetic factors. Because of the German Fertilizer Ordinance’s obligatory regulations for reduced fertilizer applications (Justiz 2017), exploiting the genetic approaches become vital for sustainable grain quality and climate-resilient agriculture at large. To this end, in this study, we evaluated the phenotypic variation, genetic control, genetic trends, and promises of genomics-assisted breeding for two important grain quality parameters, viz. Zeleny sedimentation values (ZSV) and Hagberg–Perten falling number (HFN) in a set of 372 wheat varieties that were released in previous decades and evaluated over several environments.

### Genotypic variance, genetic trends, and association among grain quality and yield reveal stable breeding for baking quality

For both ZSV and HFN, we observed a large genotypic variance and broad-sense heritability estimates—this is consistent with the previous reports (Kristensen et al. 2018; Reif et al. 2011; Würschum et al. 2016). Albeit the genotypic variance for both traits was significant, a high environmental and residual variance was observed, especially for HFN—a trait that is influenced by pre- and post-grain physiological maturity environmental conditions. Most recently, Fradgley et al. (2022) reported a large genotype-by-environment interaction for HFN in the UK winter wheat.

The effect of year-of-registration on traits under consideration provides an overall genetic trend or selection progress. Our analyses revealed, over the years, a significant increase in GY (+ 0.36%) while GPC decreased (– 0.17%) significantly. Cormier et al. (2013) observed 0.33 dt ha^−1^ increase in GY during a similar period, predominantly in French varieties. Ahlemeyer and Friedt (2011) reported a 0.34 dt ha^−1^ yearly GY increase for German varieties. More recently, Laidig et al. (2017) reported an increase in grain yield while a decrease in GPC in German variety trials. Interestingly, no statistically significant trend was observed for ZSV, HFN, and GH pointing to the likelihood that although selection pressure on GY took a toll on GPC, the traits concerning protein/gluten quality (ZSV), α-amylase activity (HFN), and grain texture (GH)—which control baking and milling quality—have sustainably been bred. While assessing long-term breeding progress in German variety trials, Laidig et al. (2017) reported positive trends for all ZSV, HFN, and GH. Our findings are also in line with Würschum et al. (2016), who reported that the sedimentation values seem to counterbalance the significant negative genetic trend for GPC while staying at consistent levels and, thus, offsetting the potential monetary losses for growers. The genetic trend for ZSV/GPC ratios further strengthens this inference (Fig. 2e).

Wheat quality traits’ negative association with grain yield is historically well known (Malloch and Newton 1934; Neatby and McCalla 1938; Oury et al. 2003; Shewry 2009). We also observed that quality traits, viz., ZSV, HFN, GPC, and GH bore a significant negative correlation with GY. ZSV and GPC showed a more pronounced negative association with GY than HFN and GH. Albeit relatively weaker, HFN showed significant correlations with the other four traits. ZSV, on the other hand, showed stronger correlations with GPC, GH, and GY. This led to an inference of a possible dissimilar genetic control of ZSV and HFN. Path analysis, nonetheless, showed no direct genetic effects of ZSV and HFN on GY. Here, GPC was the only trait having direct relationship with all other traits, including the GY. This result is usable in practical breeding programs where GPC can be used as a selection criterion for other complementary (ZSV, HFN, and GH) and antagonistic (GY) traits. In relatively early breeding generations, GPC as a trait can be scored noninvasively with high throughput via near-infrared reflectance (NIR) techniques. In addition to GPC, NIR analyses usually provide GH and grain moisture content. Thus, GPC and GH values after calibration at ~ 14% grain moisture could be suitable proxy traits to select ZSV and HFN. Also, since early generation lines are usually evaluated in micro or observation (e.g., two-row) plots and yield estimation is not possible, GPC (lower values) could be employed as one of the negative selection traits for GY when breeding for low-quality (German C class) wheat is undesirable. However, this comment on negative selection for C-class should be taken carefully since this could cause selection bias in practical breeding. For example, the stands are homogeneous if the breeding nursery is treated with fungicides, herbicides, plant growth regulators, and fertilizers. Also, in case of a few plants per plot or border effect, plants realize more nitrogen uptake which results in potentially more GPC and, consequently, deselection of potential high-yielding lines. Conversely, in untreated conditions—which is usually the case in early generations in practical breeding—higher GPC is correlated with lower disease resistance. Consequently, removing lower GPC material could also result in removing the best disease resistant material. Recently, Gogna et al. (2022) showed negative correlations of protein content with major wheat diseases such as *Septoria tritici* blotch, *Drechslera tritici-repentis*, and *Fusarium* head blight in the same GABI wheat panel.

### Genetic control of sedimentation values is simpler than falling number

The genome-wide association scan resulted in the detection of four ZSV and two HFN main-effect QTL. Comparison of QTL locations with previous mapping studies is often not possible because of the different use of: (1) marker systems (e.g., microsatellites, SNP arrays, etc.), (2) chromosome maps (e.g., genetic (cM) or physical (bp) positions), and (3) nature of mapping populations (e.g., bi-parental, synthetic, diverse released varieties, and breeding lines, etc.). Moreover, the studies on the association mapping for ZSV and HFN are limited and do not provide marker sequences, especially in winter wheats. Hence, we compare our QTL to the recent and relevant studies in the following.

Of the four QTL, three, viz. *QZsv.ipk-1A*, -*1B*, and -*1D* explained > 25% whereas the MTA representing the fourth ZSV-QTL (*QZsv.ipk-5D*) corresponding to *Pinb-D1* gene explained 11.39% of the genotypic variance. Würschum et al. (2016) also detected four medium- to large-effect QTL that explained ~ 60% of the genotypic variance. This suggests that the large-effect marker-based genetic gain on ZSV QTL could be beneficial in terms of time- and cost-efficiency. The ZSV-QTL *QZsv.ipk-1A* harbored the gene for peroxidases (POD). To our knowledge, this is the first GWAS that identified an SNP linked with POD for ZSV—most possibly because PODs can improve the physical characteristics of gluten (Geng et al. 2019): this, however, needs further genetic and functional validation in independent populations. Another ZSV-QTL *QZsv.ipk-1D* harbored markers significantly associated with *Glu-D1*—a known HMW glutenin locus that influences the gluten and eventually viscoelastic properties of wheat flour doughs (Gale 2005). In addition to *Glu-D1*, *QZsv.ipk-1D*’s MTA *BS00022768_51* explaining 26.05% of the genotypic variance corresponded to globulin—a non-gluten protein that, albeit present in minor quantities in the wheat endosperm, influences the flour processing (Gupta et al. 1992; Osborne 1907; Pence et al. 1954). Since the extent of linkage disequilibrium (the non-random association between different loci) plays a vital role in GWAS, the MTA *BS00022768_51*—because of its physical location on the long arm (412.2 Mbp)—is likely to be linked with *Glu-D1*, and, thus, may constitute the same QTL. Yu et al. (2021) recently colocalized several mixograph parameters with *Glu-D1* locus on the same physical position (412–14 Mbp). Also, Fradgley et al. (2022) identified QTL harboring *Glu-D1* to be associated with dough rheology traits in the UK winter wheat. Therefore, this locus seems to control several bread-making quality traits in wheat. *Pin-1* genes affect wheat’s milling quality by primarily controlling the grain texture (Morris 2002). *QZsv.ipk-5D* harbored an MTA *BS00000020_51* corresponding to Puroindoline-b (*Pinb-D1*). *Pinb-D1*’s association with ZSV suggests its possible role in controlling the ZSV—several previous studies have shown this gene’s association with other quality traits, including ZSV (Bhave and Morris 2008; Kristensen et al. 2018; Mohler et al. 2012). By dividing the panel based on *Pin-1* genes' profiles, we also observed highly significant differences between the soft and hard wheats for all investigated quality traits, including ZSV. As described elsewhere, breeders in Europe have—compared to *Pina-D1*—mainly tapped the genetic variation of *Pinb-D1* mutant alleles to fine-tune or maintain hardness or the grain texture. The association of this locus with ZSV further confirms the inference mentioned earlier that although GPC have reduced over the years, sustained ZSV and GH values helped sustain/improve bread-making properties. To achieve registration, in terms of GY, a line must outperform the existing check varieties (depending upon the quality class) by a certain margin and thus, the new high-yielding varieties inevitably show sustained/improved baking parameters. At this juncture, it can be safely assumed that wheat breeders seem to have exploited the ZSV/GPC ratio for which a stable and slightly positive genetic trend was observed in our study.

For HFN, seven MTA present on individual wheat chromosomes were identified. Of these seven, only two explained ~ 10% of the variance, and, thus, were designated as HFN-QTL. The *QHfn.ipk-1A*’s significant marker (*AX-94430348*; 360.5 Mbp) explained 12.74% of the genotypic variance and corresponded to the albumin protein (Fig. S8). Albumin, like globulin, is a non-gluten minor protein in wheat endosperm that has a significant bearing on wheat’s quality (Belderok et al. 2000). For example, Gupta et al. (1992) reported that dough quality is significantly related to the gluten and other monomeric proteins (e.g., globulin, and albumin). Žilić et al. (2011) identified albumins and globulins as major enzymes involved in metabolic processing. Most recently, Dallinger et al. (2023), via using a genotyping-by-sequencing platform in European winter wheat varieties, identified a QTL on chromosome 1A but at 313.5 Mbp and explaining 4% of the variation. The other HFN-QTL *QHfn.ipk-5B* (MTA: *wsnp_Ku_rep_c71565_71299640*; 618.1 Mbp) identified in this study explained 9.38% of the genotypic variance. To our knowledge, no previous reports, especially in winter wheats, identified HFN-QTL on 5B at or near 618.1 Mbp, thus, making it a novel QTL.

Taken together, our GWAS showcased ZSV’s genetic control to be less complex than HFN as its QTL showed more genotypic variance and were more significant than HFN—most possibly because of the action of known quality-related genes, e.g., *Glu-D1* and *Pinb-D1*. Nevertheless, although helpful in breeding programs for speedy selection gain, the QTL and corresponding candidate genes reported in this study need further genetic and functional validation.

### Haplotype analysis point toward suitable allelic combinations of major loci for breeding better grain quality and yield

Gluten (glutenin and gliadin) form the majority of the wheat storage proteins and the glutenin (*Glu*) loci bear a strong effect on the dough and baking quality (Gale 2005). Similarly, *Pin-1* genes are the major loci to control grain texture (Morris 2002). We investigated the effect of three HMW *Glu-1* loci (*Glu-A1*, *-B1*, and *-D1*) using candidate markers for the corresponding genes. In addition, the *Rht-D1* and *Pin-1* (*Pina-D1* and *Pinb-D1*) loci were evaluated to observe their allelic influence on the investigated traits. Of the investigated loci, *Glu-A1* alleles (Ax-null and Ax2*) did not significantly differentiate the ZSV; however, the presence of Ax2* resulted in a significant decrease in both HFN and GY. *Glu-D1* locus (Liu et al. 2008) that differentiated Dx5 + Dy10 (responsible for strong doughs) and Dx2 + Dy12 (weak doughs) subunits was significantly associated with ZSV. Würschum et al. (2016) also found a significant association of *Glu-1* loci with sedimentation values evaluated by the SDS-PAGE method. Contrary to Mohler et al. (2014) and Gooding et al. (2012), the *Rht-D1*’s effect on HFN in our study was statistically insignificant—possibly due to population size and the genetic background as the authors used bi-parental populations. In addition to *Glu-D1*, the *Pinb-D1* gene was highly significantly associated with ZSV.

In the context of using major quality loci, it is vital to observe how breeders have exploited combination of alleles (haplotypes). As stated elsewhere, new varieties—depending on the quality class—must be superior to the checks (current top-yielding varieties) in terms of GY, disease resistance, and grain quality. In this regard, our haplotype analyses shed light on some of the breeders’ “favorite” haplotypes to sustain the bread-making quality while improving grain yield in wheat (Fig. 6). For example, 36 varieties harboring Hap-1 showed significantly increased values for ZSV, HFN, and GH. At the same time, the difference of GY compared to the overall population mean was not significant. Similarly, Hap-6 showed no significant differences in GY while showing improved values for all four quality traits. The most noticeable difference for GY values was observed in Hap-8 where varieties showed 3.51 dt ha^−1^ more GY than the population mean. In contrast, all other quality traits showed, predictably, lower values—probably because both wild-type forms for *Pin-1* loci, *Glu-D1* Dx2 + Dy12, and *Glu-A1* Ax-null subunits were represented in Hap-8. In contrast, Hap-2 showed significantly decreased GY, harbored better quality subunits/alleles of major loci, and consequently significantly higher quality trait values. It should be noticed that *Glu-A1* and *-B1* subunits viz. Ax2* and Bx7^OE^ were present only in 10.56 and 6.18% and thus could be a reason for not appearing in any major haplotype: all ten haplotypes harbored Ax-null and Bx7^NE^ subunits. Similarly, from *Pin-1* loci, the *Pina-D1b* null allele was present in only 7.57% of the varieties (Fig. 5) indicating that quality profiles have mainly been shaped by *Pinb-D1* mutant alleles (*Pinb-D1b–d*). The presence of *Rht-D1a* resulted in either decreased or commensurable values for GY. However, varieties harboring *Rht-D1b* alleles showed either increased or on par GY values. Taken together, our analyses show that Bx7orBx17, Dx5 + Dy10, and *Pinb-D1b–d* subunits/alleles have been necessary for improved and sustainable breeding for GY and quality traits. Hence, these can be used in two to three combinations depending upon the target GY and quality class. Also, Hap-2 and Hap-8 may be used to breed for targeted E (high-quality elite) and C (cookies or stock feed) class GY groups, respectively.

### The prospects of predictive breeding for sedimentation values and falling number in applied wheat breeding programs

We employed high-density SNP arrays, SSRs, and diagnostic markers with multi-environment robust phenotypic data for GWAS. However, the genotypic variance imparted by the identified MTA amounted to roughly 40% for ZSV and HFN. Hence, the remaining ~ 60% unexplained genotypic variance point to many small-effect maker loci, each explaining less than 10% genotypic variance. The prospects of genome-wide prediction—to predict the total genetic value of a trait based on all maker loci irrespective of their effect size—could be utilized (Meuwissen et al. 2001). In this study, based on five different models, predictive accuracies for ZSV and HFN suggested that these traits could be used for efficient genomic selection. Reports on genomic selection for both ZSV and HFN are scarce. Our results align with recent reports of Würschum et al. (2016) and Kristensen et al. (2018), who, while studying European wheat varieties/lines, reported high predictive abilities for ZSV and HFN. Here, it is worth noting that RKHSR—a model used to assess epistatic interactions among loci—did not outperform other models that primarily exploit the additive effects. This is in line with Würschum et al. (2016) where the authors did not find any significant epistatic QTL that could be exploited in marker-assisted breeding. Most recently, Schwarzwälder et al. (2022) reported that (1) selection on sedimentation values could help improve the baking volume, and (2) lines possessing high per se quality traits should help develop good hybrids: this is because most of the quality traits are additive, and there exists a high correlation between the mid-parent and hybrid performance. In this context, the mean prediction accuracies suggest that genomic selection could help select the lines with better genetic merit for ZSV and HFN. This could help achieve better genetic gains in both line and hybrid breeding programs.

### Supplementary Information

Below is the link to the electronic supplementary material.

Supplementary file 1 (PDF 1178 KB)

Supplementary file 2 (XLSX 97 KB)

## Data Availability

The authors declare that all data are contained within the paper and supplementary information. The phenotypic and marker datasets were published in Gogna et al (2022).
