# Supplementary material for: Genetic control and prospects of predictive breeding for European winter wheat’s Zeleny sedimentation values and Hagberg-Perten falling number
Source: Theor Appl Genet. 2023 Oct 24;136(11):229. doi: 10.1007/s00122-023-04450-7 (PMC10598174; doi:10.1007/s00122-023-04450-7)
Supplement: Supplementary file 1 — (PDF 1178 KB) [file 122_2023_4450_MOESM1_ESM.pdf]

Article type: **Research article**

# Genetic control and prospects of predictive breeding for European winter wheat's Zeleny sedimentation values and Hagberg-Perten falling number

Quddoos H. Muqaddasi [1,7,*], Roop Kamal Muqaddasi [2], Erhard Ebmeyer [3], Viktor Korzun [4], Odile Argillier [5], Vilson Mirdita [1], Jochen C. Reif [2], Martin W. Ganal [6], and Marion S. Röder [2]

[1] European Wheat Breeding Center, BASF Agricultural Solutions GmbH, Am Schwabeplan 8, 06466 Stadt Seeland OT Gatersleben, Germany
[2] Leibniz Institute of Plant Genetics and Crop Plant Research (IPK), Corrensstraße 3, 06466 Stadt Seeland OT Gatersleben, Germany
[3] KWS Lochow GmbH, 29303 Bergen, Germany
[4] KWS SAAT SE & Co. KGaA, 37574 Einbeck, Germany
[5] Syngenta, 28008 Chartres Cedex, France
[6] TraitGenetics GmbH, Am Schwabeplan 1b, 06466 Stadt Seeland OT Gatersleben, Germany
[7] Present address: KWS SAAT SE & Co. KGaA, 37574 Einbeck, Germany
* Corresponding author: quddoos.muqaddasi@kws.com

## Supplementary figures

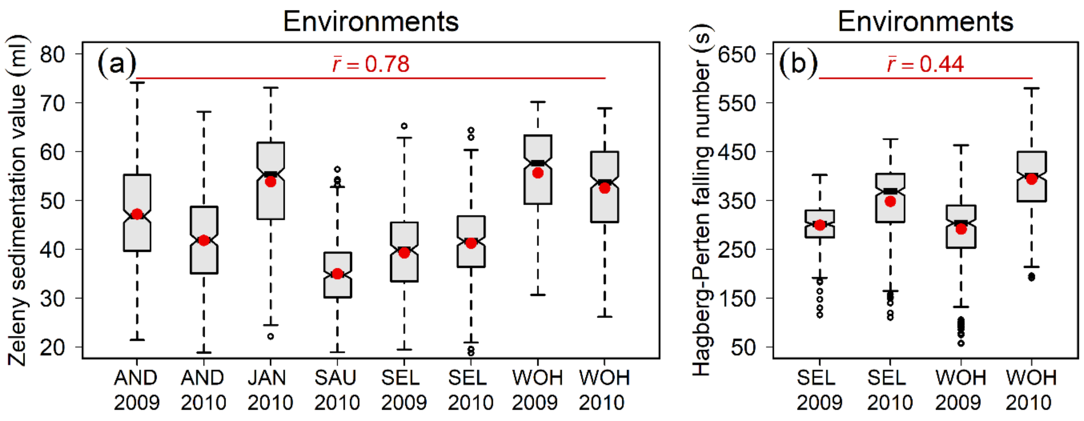


**Figure S1.** Environment specific phenotypic distribution of the investigated traits (**a**) Zeleny sedimentation values and (**b**) Hargberg-Perten falling number in a panel of 372 European wheat varieties. x-axis represent the environments (i.e. location x year combinations) while $\bar{r}$ denotes the average trait correlation calculated across environments by performing Fisher's $z$ transformation.

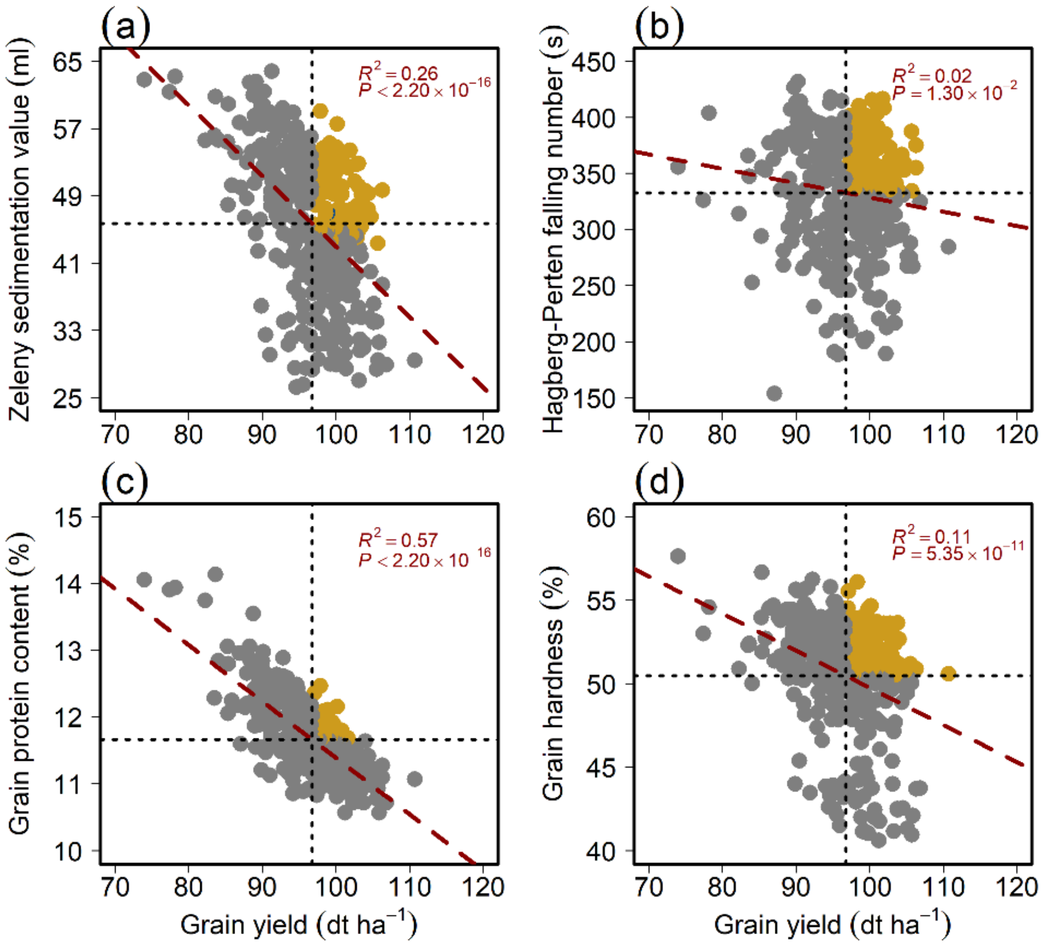


**Figure S2.** Association of wheat's grain yield with four quality traits namely **(a)** Zeleny sedimentation values, **(b)** Hagberg-Perten falling number, **(c)** grain protein content, and **(d)** grain hardness based on their BLUEs calculated across environments. $R^2$ and $P$ denote the multiple squared regression coefficient and its respective significance. The red dashed line represent the linear regression line whereas the golden dots represent the above average values for both traits in each scatter plot.

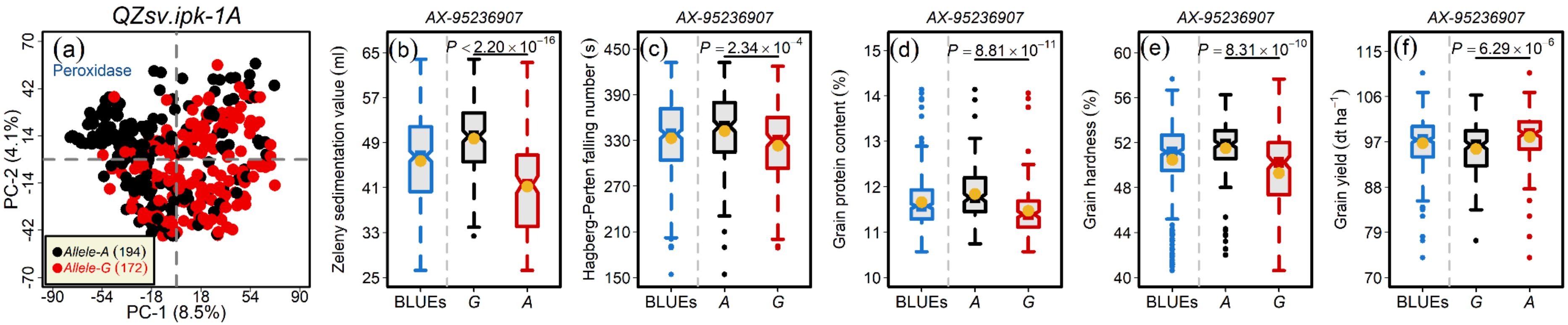


**Figure S3.** Scatterplot and boxplots showing the distribution of the (**a**) identified *QZsv.ipk-1A* QTL for Zeleny sedimentation value represented by the marker *AX-95236907* as a function of first two principal components based on high-quality marker genotypes, and the allelic influence of *AX-95236907* on (**b**) Zeleny sedimentation value, (**c**) Hagberg-Perten falling number, (**d**) Grain protein content, (**e**) grain hardness, and (**f**) grain yield. The blue, black, and red boxplots show the overall genotypic values (BLUEs) of varieties, varieties harboring the major (reference) allele and the minor (alternate) allele, respectively. The annotated function of the corresponding gene for *AX-95236907* is Peroxidase, as highlighted blue in **(a)**.

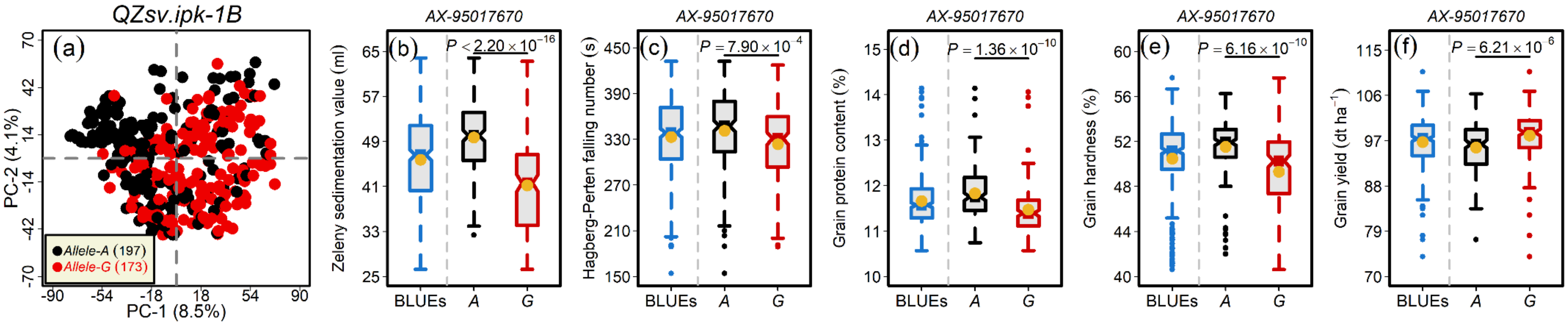


**Figure S4.** Scatterplot and boxplots showing the distribution of the (**a**) identified *QZsv.ipk-1B* for Zeleny sedimentation value represented by the marker *AX-95017670* as a function of first two principal components based on high-quality marker genotypes, and the allelic influence of *AX-95017670* on (**b**) Zeleny sedimentation value, (**c**) Hagberg-Perten falling number, (**d**) Grain protein content, (**e**) grain hardness, and (**f**) grain yield. The blue, black, and red boxplots show the overall genotypic values (BLUEs)of varieties, varieties harboring the major (reference) and the minor (alternate) allele, respectively.

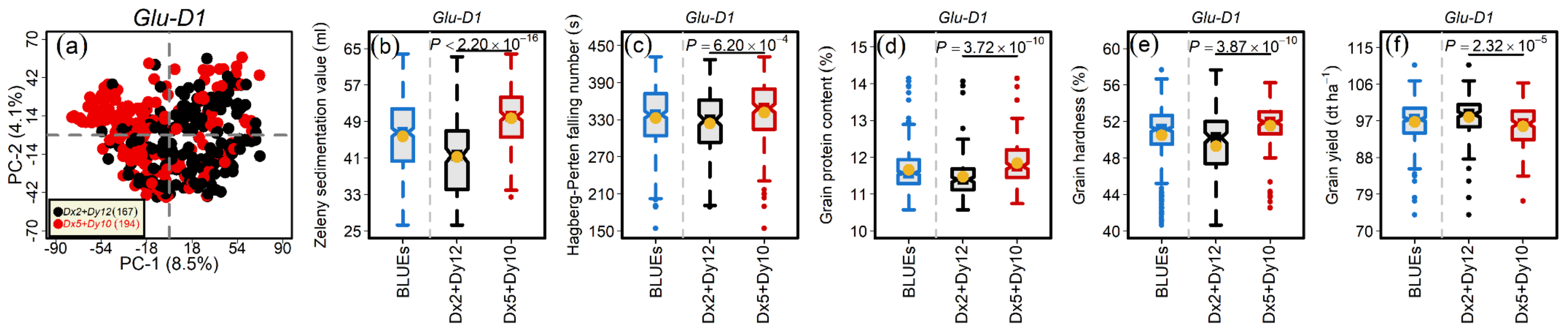


**Figure S5.** Scatterplot and boxplots showing the distribution of the (**a**) identified *QZsv.ipk-1D* for Zeleny sedimentation value represented by the marker UMN25 and UMN26 for *Glu-D1* as a function of first two principal components based on high-quality marker genotypes, and the allelic influence of *Glu-D1* on (**b**) Zeleny sedimentation value, (**c**) Hagberg-Perten falling number, (**d**) Grain protein content, (**e**) grain hardness, and (**f**) grain yield. The blue, black, and red boxplots show the overall genotypic values (BLUEs) of varieties, varieties harboring the major (reference) and the minor (alternate) allele, respectively.

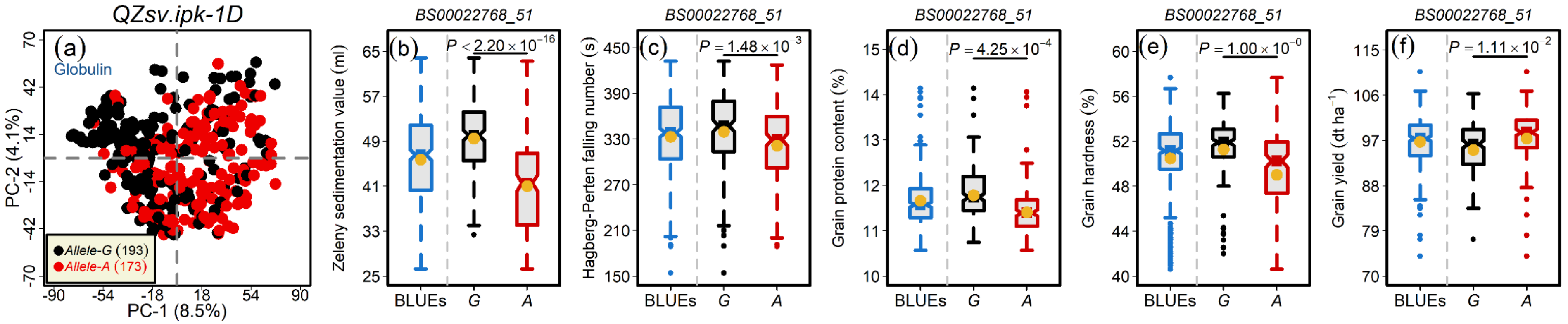


**Figure S6.** Scatterplot and boxplots showing the distribution of the (**a**) identified *QZsv.ipk-1D* for Zeleny sedimentation value represented by the marker *BS00022768_51* as a function of first two principal components based on high-quality marker genotypes, and the allelic influence of *BS00022768_51* on (**b**) Zeleny sedimentation value, (**c**) Hagberg-Perten falling number, (**d**) Grain protein content, (**e**) grain hardness, and (**f**) grain yield. The blue, black, and red boxplots show the overall genotypic values (BLUEs) of varieties, varieties harboring the major (reference) and the minor (alternate) allele, respectively. The annotated function of the corresponding gene for *BS00022768_51* is Globulin, as highlighted blue in **(a)**.

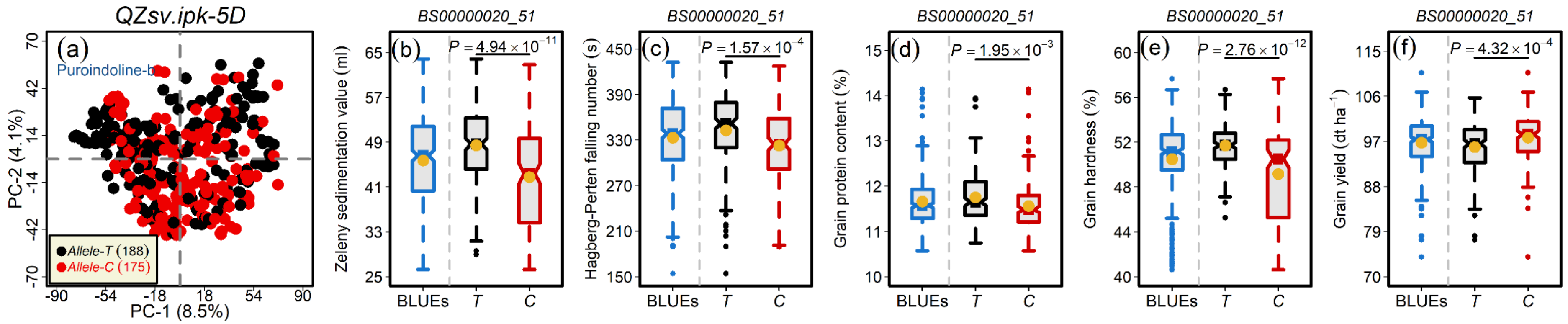


**Figure S7.** Scatterplot and boxplots showing the distribution of the (**a**) identified *QZsv.ipk-5D* for Zeleny sedimentation value represented by the marker *BS00000020_51* as a function of first two principal components based on high-quality marker genotypes, and the allelic influence of *BS00000020_51* on (**b**) Zeleny sedimentation value, (**c**) Hagberg-Perten falling number, (**d**) Grain protein content, (**e**) grain hardness, and (**f**) grain yield. The blue, black, and red boxplots show the overall genotypic values (BLUEs) of varieties, varieties harboring the major (reference) and the minor (alternate) allele, respectively. The annotated function of the corresponding gene for *BS00000020_51* is Puroindoline-b, as highlighted blue in **(a)**.

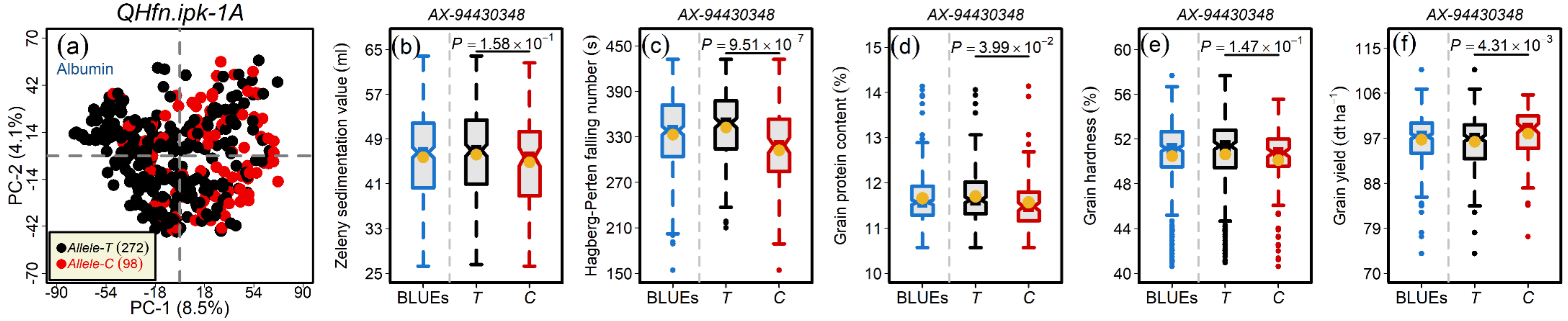


**Figure S8.** Scatterplot and boxplots showing the distribution of the (**a**) identified *QHfn.ipk-1A* for Hagberg-Perten falling number represented by the marker *AX-94430348* as a function of first two principal components based on high-quality marker genotypes, and the allelic influence of *AX-94430348* on (**b**) Zeleny sedimentation value, (**c**) Hagberg-Perten falling number, (**d**) Grain protein content, (**e**) grain hardness, and (**f**) grain yield. The blue, black, and red boxplots show the overall genotypic values (BLUEs) of varieties, varieties harboring the major (reference) and the minor (alternate) allele, respectively. The annotated function of the corresponding gene for *AX*-94430348 is Albumin-2 protein, as highlighted blue in **(a)**.

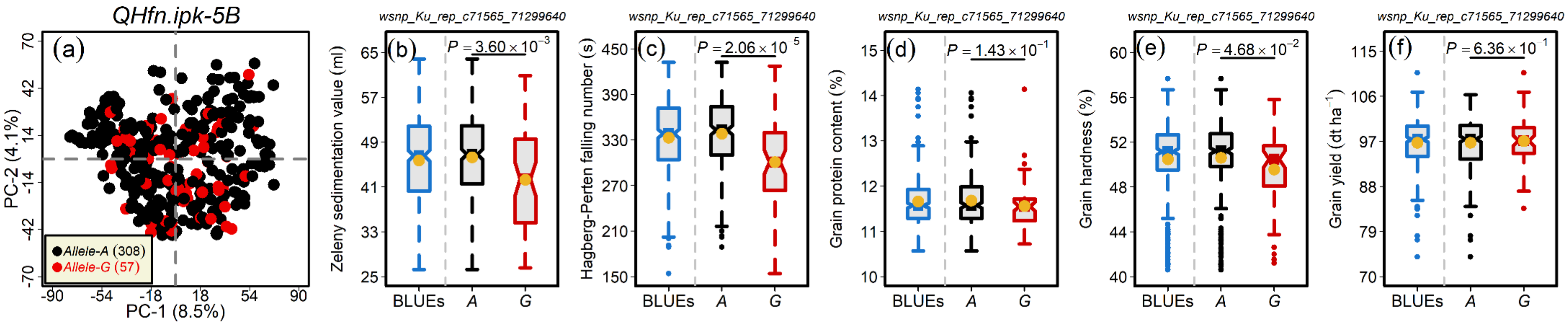


**Figure S9.** Scatterplot and boxplots showing the distribution of the (**a**) identified *QHfn.ipk-5B* for Hagberg-Perten falling number represented by the marker *wsnp_Ku_rep_c71565_71299640* as a function of first two principal components based on high-quality marker genotypes, and the allelic influence of *wsnp_Ku_rep_c71565_71299640* on (**b**) Zeleny sedimentation value, (**c**) Hagberg-Perten falling number, (**d**) Grain protein content, (**e**) grain hardness, and (**f**) grain yield. The blue, black, and red boxplots show the overall genotypic values (BLUEs) of varieties, varieties harboring the major (reference) and the minor (alternate) allele, respectively.

- - -
